# Clinical long-term and patient-reported outcomes of dental implants in oral cancer patients

**DOI:** 10.1186/s40729-021-00373-4

**Published:** 2021-07-13

**Authors:** Eik Schiegnitz, Lena Katharina Müller, Keyvan Sagheb, Lisa Theis, Vahide Cagiran, Peer W. Kämmerer, Joachim Wegener, Wilfried Wagner, Bilal Al-Nawas

**Affiliations:** 1 Department of Oral and Maxillofacial Surgery, Plastic Surgery, University Medical Centre, Johannes Gutenberg-University, Mainz, Germany; 2 Department of Prosthodontics, University Medical Centre, Johannes Gutenberg-University, Mainz, Germany

**Keywords:** Long-term survival, Dental implant, Augmented bone, Native bone, Irradiation, Alveolar ridge reconstruction, Oral cancer

## Abstract

**Background and purpose:**

The aim of this clinical study was to investigate the clinical long-term and patient-reported outcome of dental implants in patients with oral cancer. In addition, analysis of the influence of radiation therapy, timing of implant insertion, and augmentation procedures on implant survival was performed.

**Material and methods:**

This retrospective study investigated the clinical outcome of 711 dental implants in 164 oral cancer patients, inserted by experienced surgeons of the Department of Oral and Maxillofacial Surgery, University Medical Center Mainz, Germany. Oral health-related quality of life (OHRQoL) was evaluated.

**Results:**

Cumulative 5-year and 10-year implant survival rates for all included implants were 87.3% and 80.0%. Implants placed straight after ablative surgery (primary implant placement) and implants placed after completing the oncologic treatment (secondary implant placement) showed a comparable implant survival (92.5% vs. 89.5%; p = 0.635). Irradiation therapy had no significant influence on implant survival of secondary placed implants (p = 0.929). However, regarding implant site (native bone vs. augmented bone) and radiation therapy (non-irradiated bone vs. irradiated bone), implants inserted in irradiated bone that received augmentation procedures showed a statistically significant lower implant survival (p < 0.001). Patients reported a distinct improvement in OHRQoL.

**Conclusions:**

Promising long-term survival rates of dental implants in patients after treatment of oral cancer were seen. In addition, patients benefit in form of an improved OHRQoL. However, bone augmentation procedures in irradiated bone may result in an impaired implants’ prognosis.

**Supplementary Information:**

The online version contains supplementary material available at 10.1186/s40729-021-00373-4.

## Introduction

Every year more than 650,000 people around the globe are affected by head and neck cancer, which causes 330,000 deaths annually [1]. Oral cancer alone accounts 2–4% of all cancer worldwide with elevated prevalences in India, Pakistan, and southeast Asia [2–4]. Over the past decades, a significant improvement in overall 5-year survival rates from 54.7% in 1992–1996 to 65.9% in 2002–2006 was observed, with the greatest improvements in patients aged 15–64 years with oral cavity cancer [5]. Actual treatment concepts of head and neck cancer include a combination of surgery and radiotherapy, based on the degree of severity of the disease. All treatment concepts result in either surgical defect together with a fragile and tender mucosa, an altered orofacial anatomy and xerostomia.

For functional, aesthetic, and physiognomic rehabilitation of oral cancer-related problems, the insertion of dental implants may be indicated. Several studies have shown, that the insertion of dental implants can improve the oral health-related quality of life [6, 7] and offers benefits when compared to conventional tissue-born dental prosthesis [8]. For this reason, the option of inserting implants in patients with a history of oral cancer and irradiation in this area should be verified in each individual patient case. However, the oral rehabilitation of patients irradiated in the head and neck region is complex and many parameters, such as radiation dose, timing of radiation, site of implant placement (mandibula vs. maxilla), vascularized free flaps, non-vascular bone grafts, smoking habits, and oral hygiene should be considered.

There have been numerous studies regarding the optimal timing of implant placement regarding implant survival rates. Traditionally, implants were placed after completion of oncological treatment (secondary implant placement), but over the last years various studies suggest higher survival rates for implants placed during ablative surgery (primary placed), which is furthermore accompanied by earlier prosthetic rehabilitation [9–13]. A meta-analysis of the data confirmed slightly higher survival rates for primary placed implants compared to secondary placed implants [14]. In contrast, a review by Nooh et al. found no significant difference between primary or secondary placed implants [15].

In general, radiation therapy may be a variable negatively affecting long-term survival of dental implants [16], although existing studies remain partially inconsistent [17]. A meta-analysis by Chambrone et al. showed a higher risk for implant failure in radiated patients compared to non-irradiated patients [18]. However, a recent study showed similar survival rates in both, patients who received radiation therapy and those who did not receive radiation therapy during their oncological treatment [19]. It should be mentioned that this study had very strict eligibility criteria and therefore other variables influencing long-term implant survival were excluded. Smokers were excluded for participation and soft-tissue augmentations were performed in case of insufficient soft-tissue conditions. Smoking is known to negatively influence endosseous implant survival aside from other variables such as radiation [20].

The radiation dose applied to the tumor volume, which is evaluated in many studies, may differentiate from the implant-bed specific dose. Wide ranges of implant-bed-specific mean radiation dose were found from 3.2 Gy to 71.4 Gy, indicating that a precise evaluation of implant-bed specific radiation doses should be considered if dose-dependent effects of radiotherapy on long-term survival of dental implants are evaluated [21]. A meta-analysis showed better survival rates for primary placed implants not having received radiotherapy compared to those who received radiotherapy [14]. Regarding the timepoint of radiation, similar failure rates were evaluated for implants placed after radiotherapy compared to those placed before radiotherapy [22].

Ablative surgery of oral tumors may generate the need for autologous bone augmentation. Attia et al. showed excellent clinical outcomes of dental implant placement in free fibula flaps in a retrospective study. Therefore, oral rehabilitation with dental implants in free fibula flaps may be considered as a safe procedure [23]. Chang et al. recently presented a case, in which a free fibula flap was placed as an onlay graft after marginal mandibulectomy in order to increase the height of the alveolar ridge for endosseal implantation [24]. Endosseous implant survival in oncological patients who received vascularized or non-vascularized autologous bone grafts is promising; however, the available data is inconsistent and implant survival varies highly among the studies [25].

Overall, implant placement in oncology patients is a predictable procedure. A recent review of the literature showed a 97.16% success rate in healthy patients vs. a success rate of 93.02% in oncological patients [26]. Although, a large number of confounding parameters should be considered. Thus, the aim of the retrospective study was to investigate prognostic parameters for the clinical and patient-reported long-term outcome of dental implants in patients with intraoral squamous cell carcinoma. The null hypothesis was that dental implants in the irradiated and augmented area have a worse long-term outcome.

## Material and methods

### Patient selection

In the present study, records of patients with intraoral squamous cell carcinoma and cancer-related placement of dental implants were included. The patients were treated between March 1996 and December 2014 in the Department of Oral and Maxillofacial Surgery, University Medical Center Mainz, Germany. Ethical approval was obtained from the ethical committee of Rhineland-Palatinate, Germany (Registration number: 2020-14895, Landesärztekammer Rheinland-Pfalz). The study was conducted in accordance with the Helsinki Declaration of 1975 as revised in 2000. The present retrospective study included 164 oral cancer patients with 711 dental implants. Mean age of the patients was 67.3 years. One hundred ten patients were males (67%) and 54 females (33%). According to the TNM classification the patients included had the following classification: T1 (n = 53), T2 (n = 62), T3 (n = 11), T4 (n = 38), N0 (n = 101), N1 (n = 27), N2 (n = 34), N3 (n = 2), M0 (n = 163), and M1 (n = 1). According to the timing of dental implant insertion, 117 patients received dental implants after completing the oncologic treatment (secondary implant placement). In 47 patients, implants were placed straight after ablative surgery (primary implant placement).

Standardized protocols for dental implant insertion were followed for all patients who received radiation therapy. A prophylactic antibiosis (amoxicillin/clavulanic acid 875/125 mg or amoxycillin 1000 mg) was started prior surgery and continued for 3 days. In case of allergy or intolerance to penicillin, clindamycin 600 mg was prescribed. After preparation of a muco-periosteal flap, implants were inserted according to the manufacturers’ surgical protocol. After insertion, implants were left for submerged healing for at least 3 months. Radiation therapy was performed in 72 subjects (44%), and 86 subjects (52%) did not receive radiation. In 6 patients (4%), no information regarding radiotherapy could be evaluated retrospectively. In case of radiation therapy, normally a dose of 64 Gy in 30 fractions was delivered to the primary tumor region in form of intensity-modulated radiation therapy (IMRT). Regarding augmentation techniques, 31 implants were inserted in fibula grafts and 42 implants in iliac crest bone graft. Four implants were inserted in combination with guided bone generation, 8 implants were inserted in combination with sinuslift procedures, and 13 implants were inserted in combination with a distraction osteogenesis. In two cases, fibula and iliac bone grafts were combined. Regarding radiation therapy and the location of the implants placed, 273 implants (38.4%) were inserted in native/non-irradiated bone, 316 implants (44.4%) in native-irradiated bone, 45 implants (6.3%) in augmented/non-irradiated bone, and 50 implants (7.0%) in radiated/augmented bone. For 27 implants (3.8%) implant site and irradiation could retrospectively not be evaluated. A total of 234 implants were inserted into the maxilla and 477 implants were placed into the mandibula. One hundred sixty-eight implants were narrow diameter implants (diameter ≤ 3.5 mm) and 537 implants were standard diameter implants (diameter > 3.5 mm). For 5 implants, implant diameter was not traceable. Thirty implants were short dental implants (length ≤ 6 mm) and 674 implants were standard length dental implants (length > 6 mm). For 6 implants, implant length was not traceable. The used prosthetic superstructures for edentulous patients were mainly bars. For partially edentulous patients, fixed dental prothesis and single crowns were mainly used. Thirty-five patients with 165 implants presented for clinical follow-up examination. In these patients, the width of the attached gingiva, plaque index, and bleeding index were evaluated. Radiography via orthopantomogram or intraoral radiography was performed at the time of examination. The up-to-date and the postoperative radiograph were examined to evaluate the distance from the implant-abutment periphery to the apex of the implant as described before [27]. With limitations, this method has shown to be robust for this purpose [28, 29].

### Inclusion criteria

1. Patients with intraoral squamous cell carcinoma, who were treated in the Department of Oral and Maxillofacial Surgery, University Medical Center Mainz, Germany, and received dental implants between March 1996 and December 2014

2. Patient age ≥ 18 years.

### Exclusion criteria

1. Patients without detailed baseline medical data.

### Oral health-related quality of life

For assessment of oral health-related quality of life (OHRQoL), the questionnaire from Mueller et al. was used [7]. Thirty-five patients with 165 dental implants completed Mueller et al. questionnaires.

### Statistical analysis

Statistical analysis was performed using SPSS (version 23 IBM®, USA) and implant-related data were calculated. The null hypothesis was that dental implants in the irradiated and augmented area have a worse long-term outcome. No adjustment to multiple testing was performed. The Kaplan–Meier survival function was used for the description of survival rates. To analyze the statistical difference of prognostic factors, a log-rank test was performed.

## Results

### Survival rates

For all included patients and implants, mean follow-up was 45 ± 40 months (range 0 to 227 months). During follow-up, 70 implants failed, resulting in a survival rate of 90.2%. The reasons for implant loss were primary failures (n = 6), periimplant disease (n = 42), relapse of the tumor (n = 17), and osteoradionecrosis (n = 3). Cumulative 5-year and 10-year implant survival rates were 87.3% and 80.0%. Concerning implant diameter, narrow diameter implants (diameter ≤ 3.5 mm) showed a comparable implant survival rate than standard diameter implants (diameter > 3.5 mm, 88.7% vs. 90.5%, p = 0.316). Short dental implants (length ≤ 6 mm) showed a trend toward a lower implants’ survival compared to standard length dental implants (length > 6 mm, 86.7% vs. 90.2%, p = 0.062). However, as only 30 short implants were inserted, validity of this result remains limited. Implant survival rate in the upper jaw was significantly higher than in the lower jaw (94.0% vs. 88.3%, p = 0.027).

### Timing of implant insertion

**Fig. 1:**
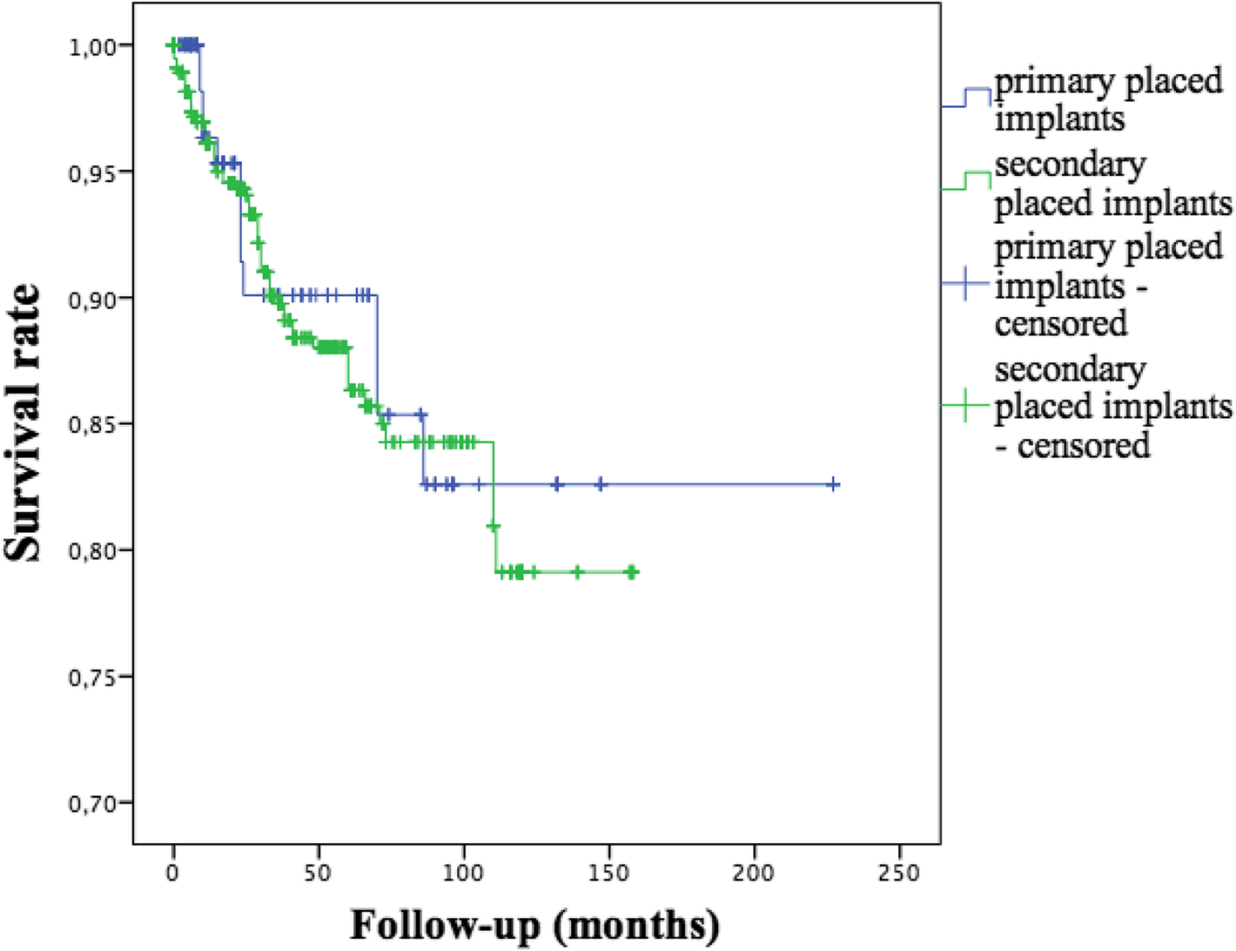
Survival rates of primary and secondary placed implants (p = 0.635)

According to the timing of dental implant insertion, survival rate of primary placed implants after ablative surgery was 92.5% (n = 159 dental implants, 12 lost implants) with a mean follow-up of 42 ± 49 months. Survival rate of dental implants placed after completing oncologic treatment (secondary implant placement) was 89.5% (n = 552 dental implants, 58 lost implants) with a mean follow-up of 46 ± 37 months (p = 0.635, Fig. 1). Here, the mean time between completion of cancer therapy and implant placement was 43.6 months.

### Radiation and bone augmentation

**Fig. 2:**
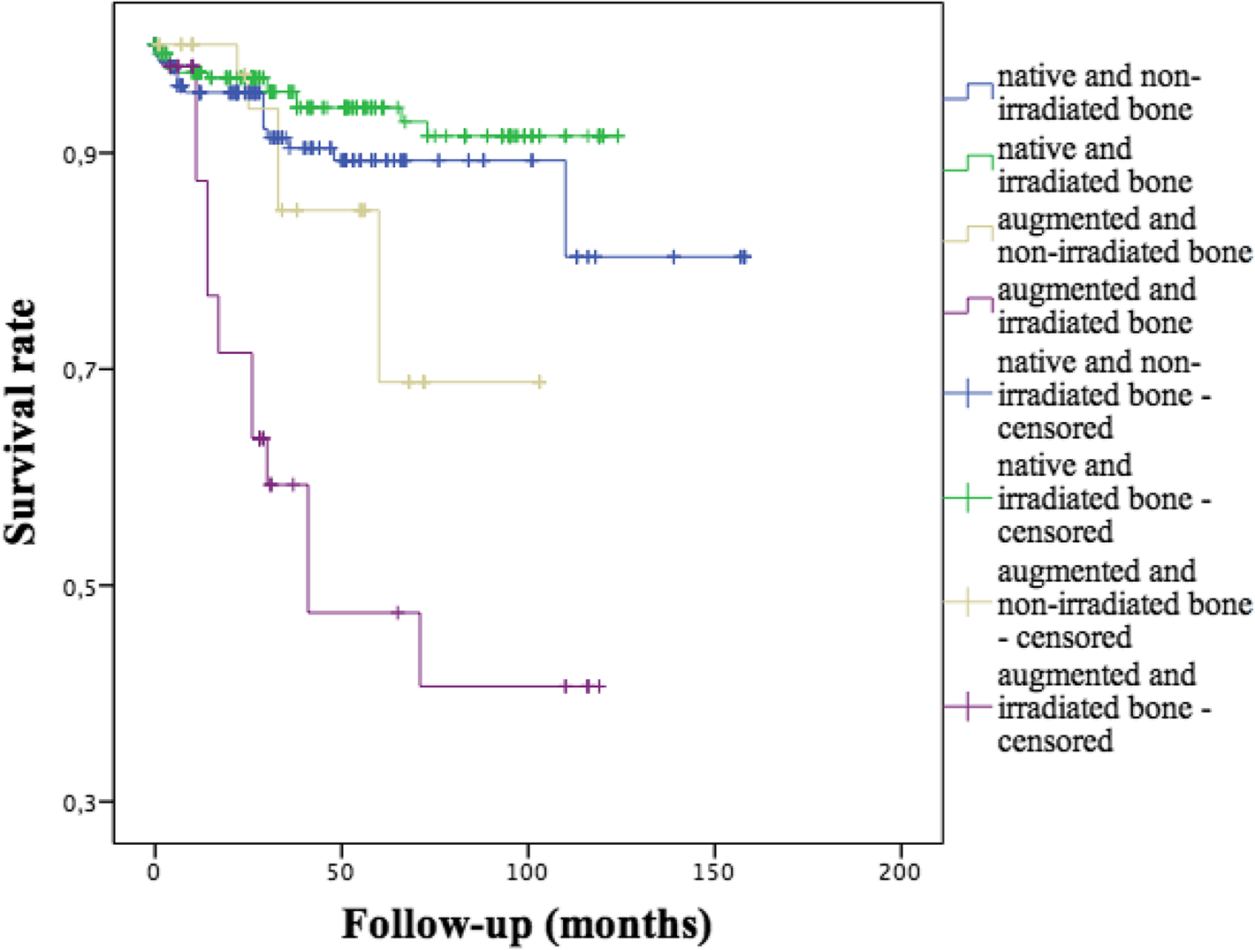
The influence of the implant site (native bone vs. augmented bone) and irradiation (non-irradiated bone vs. irradiated bone) on long-term survival (p < 0.001).

For secondary placed implants, the survival rate of implants inserted in irradiated bone was 89.3% (n = 291 implants, 31 implants lost) and 89.6% for implants inserted in non-irradiated bone (n = 240 implants, 25 implants lost) without significant differences (p = 0.929). For 21 implants, information about radiation therapy was not available. Survival rates for implants inserted in native and non-irradiated bone were 91.3%, for implants inserted in native and irradiated bone 94.6%, for implants inserted in augmented and non-irradiated bone 82.2% and for implants inserted in augmented and irradiated bone 64.0%. Implants placed into augmented and irradiated bone showed a significant worse long-term survival (p < 0.001, Fig. 2).

### Clinical follow-up and oral health-related quality of life

**Fig. 3:**
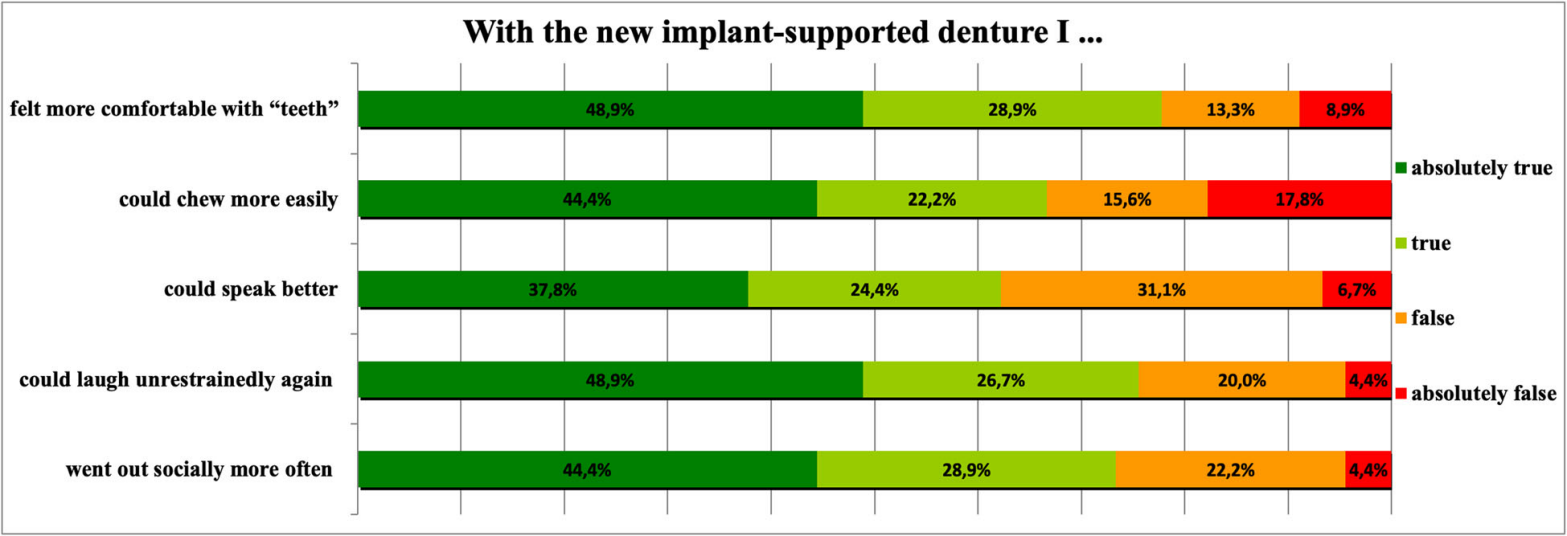
Responses to pre-worded statements after Mueller et al. [7] concerning changes in life effected by implant-supported prostheses (n = 165 implants)

The plaque index showed that 60.6% of the implants had a satisfactory degree of oral hygiene (grade 0 and 1). Seventy-nine percent of the implants showed a satisfactory bleeding index (grade 0 and 1). Mean level of attached gingiva was 0.86 ± 1 mm (range 0 to 4 mm). Mean marginal bone loss was 0.98 ± 1 mm (range 0 to 6.5 mm). Concerning the questionnaire from Mueller et al. [7] the majority of the patients (78%) felt more comfortable with their implant-supported dentures (Fig. 3). 66% of the patients reported improved chewing ability. Better speech was stated by 62% of the patients and 76% claimed that they had again begun to laugh unrestrainedly. Seventy-four percent of the interviewed patients felt that they were able to socialize more often.

## Discussion

Overall implant survival rate in oral cancer patients was 90.2% in this study, which is consistent with published data [12, 30–36]. Thus, dental implants are a safe method to achieve oral rehabilitation in patients with a history of oral cancer although several factors may influence long-term survival. In our study, irradiation had no significant influence on implant survival. In the literature were controversial results published on this topic. A meta-analysis of the literature of the years 2007 to 2013 showed no statistically significant difference in implant survival between non-irradiated native bone and irradiated native bone. In contrast, meta-analysis of the literature of the years 1990–2006 showed a significant difference in implant survival between non-irradiated and irradiated patients with a higher implant survival in the non-irradiated bone [8]. Nobrega et al. included 40 studies with 2220 participants and 9231 dental implants in their systematic review [37]. The survival rates of the studies showed a survival rate of 84.3% for implants inserted in irradiated bone tissue. The meta-analysis revealed statistically significant differences between success rates of implants placed in irradiated areas and those of implants placed in non-irradiated areas. Koudougou et al. investigated the outcomes of implants placed during ablative surgery in patients with head and neck cancer who underwent postoperative radiotherapy [38]. Implants inserted after radiation therapy and implants placed in reconstructed jaws were excluded in this review. The survival rate with postimplantation radiotherapy was 89.6% versus 98.6% in patients with no additional radiation. The overall success of implant-retained overdenture in patients with radiotherapy performed postimplantation was 67.4% versus 93.1% in patients with implant surgery that was carried out 1 year after the completion of radiation therapy. Five cases of osteoradionecrosis of the jaw were described. The authors concluded, that the outcomes for implant survival rates seem to be positive for irradiated implants. In conclusion, dental implants installed in the irradiated jaw show high survival rates, but strict monitoring is needed to prevent complications, thereby reducing possible failures.

Concerning the implantation timepoint in oral cancer patients the cumulative survival rate in our study was 89.5% for secondary placed dental implants after completing tumor therapy and 92.5% for dental implants inserted during tumor therapy. Therefore, the results of this work lead to the conclusion that primary implantation during tumor resection has a comparatively positive prognosis compared to secondary implantation. Regarding the literature, there is little consensus on the optimal time interval between tumor therapy, especially radiation, and implantation. Colella et al. found no difference regarding implant pre- and post-radiotherapy [22], whereas Granström et al. suggest to perform dental implantation procedure 6–18 months after radiation [39]. Sammartino et al. indicated that a time greater than 12 months as interval between last irradiation and implant placement seems not to promote better clinical results [40]. A meta-analysis by Claudy et al. showed that placing implants in bone within a period shorter than 12 months after radiotherapy may result in a higher risk of failure [41]. The authors therefore suggested that a waiting period of 12 months may reduce the risk of dental implant failure. In a recent review, a literature search for studies dealing with primary and/or secondary implant placement was performed [9]. The primary outcome was 5-year implant survival. Both primary and secondary implant placement indicated promising overall implant survival ratios with a higher pooled 5-year implant survival rate for primary implant placement than secondary placed implants. Primary implant placement was linked to earlier prosthetic rehabilitation after tumor surgery. The authors concluded that patients with oral cancer greatly benefit from, preferably primary placed, dental implants in their prosthetic rehabilitation.

In our study, a significant influence of the implant site (native bone vs. augmented bone) and radiation therapy (non-irradiated bone vs. irradiated bone) on long-term survival was seen. In this context, implant survival rate in native and non-irradiated bone was surprisingly lower than survival rate in native and irradiated bone. This could be explained due to a more conservative approach perioperatively for example with longer submerged healing times. In our results, implants in irradiated and augmented bone showed the lowest implant survival rates. These findings are confirmed in the international literature. A meta-analysis of 8 studies showed higher implant survival in the irradiated native bone compared to the irradiated grafted bone [8]. In a further recent meta-analysis, dental implant survival in patients undergoing vascularized maxillary or mandibular reconstruction was analyzed through a systematic review of the literature [42]. Weighted implant survival was 92.2% with a median follow-up of 36 months. Dental implants without radiotherapy exposure showed better survival rates than those exposed to radiation. Meta-analyses indicated that radiation significantly increased the risk of implant failure. Implants placed before radiotherapy trended toward better survival. The authors concluded that radiotherapy in patients undergoing vascularized maxillary or mandibular reconstruction adversely impacted dental implant outcomes and that implants placed before radiotherapy may demonstrate superior survival than implants placed after. The lower implant survival in the augmented bone may be explained by differences in bone quality, bone volume, and revascularization compared to the native bone. Therefore, implant placement in native bone should be preferred in irradiated patients.

In our study, no significant influence of implant diameter and length on long-term dental implant survival was observed. When evaluating the literature with regards to implant diameter, our data is consistent with the literature [30, 33, 43, 44]. A recent review investigated the literature on short dental implants and examined whether they are a viable definitive treatment option for rehabilitating cancer patients with deficient bone [45]. The results showed that short implants can achieve results similar to those of longer implants in augmented bone and offer a treatment alternative in the rehabilitation of patients with cancer that could reduce the need for invasive surgery and associated morbidity and be safer and more economical.

Our study showed an improved OHRQoL after implant-prosthetic rehabilitation. The majority of the cancer patients felt more comfortable with their implant-supported dentures, reported improved chewing ability, a better speech, and claimed that they had again begun to laugh unrestrainedly. In addition, most patients felt that they were able to socialize more often compared to before dental implants were inserted. These are all essential components for an improved quality of life. Therefore, oral cancer patients benefit from implant-prosthetic rehabilitation and such treatment concepts should always be individually discussed with the cancer patient in case of missing teeth.

## Conclusion

In conclusion, our results show that a successful and safe rehabilitation of the irradiated oral cancer patient with high implant survival rates is possible, for either secondary as well as primary placed implants. However, bone augmentation in the irradiated jaw should be considered as a negative prognostic factor. This demonstrates that an implantation in the native bone should be preferred among irradiated tumor patients.

## Supplementary Information

**Additional file 1.**

## Data Availability

Not applicable

## Abbreviations

OHRQoL: Oral health-related quality of life
