# Supplementary material for: Clinical long-term and patient-reported outcomes of dental implants in oral cancer patients
Source: Int J Implant Dent. 2021 Jul 13;7:93. doi: 10.1186/s40729-021-00373-4 (PMC8276905; doi:10.1186/s40729-021-00373-4)
Supplement: Supplementary file 1 — Additional file 1. [file 40729_2021_373_MOESM1_ESM.pdf]

Überlebenstabelle

	Zeit	Status	Kumulierter Anteil Überlebender zum Zeitpunkt		Anzahl der kumulativen Ereignisse	Anzahl der verbliebenen Fälle
			Schätzer	Standardfehler		
1	,000	1	.	.	1	710
2	,000	1	.	.	2	709
3	,000	1	,996	,002	3	708
4	,000	0	.	.	3	707
5	,000	0	.	.	3	706
6	,000	0	.	.	3	705
7	,000	0	.	.	3	704
8	,000	0	.	.	3	703
9	,000	0	.	.	3	702
10	,000	0	.	.	3	701
11	,000	0	.	.	3	700
12	,000	0	.	.	3	699
13	,000	0	.	.	3	698
14	,000	0	.	.	3	697
15	,000	0	.	.	3	696
16	,000	0	.	.	3	695
17	,000	0	.	.	3	694
18	,000	0	.	.	3	693
19	,000	0	.	.	3	692
20	,000	0	.	.	3	691
21	,000	0	.	.	3	690
22	,000	0	.	.	3	689
23	1,000	1	.	.	4	688
24	1,000	1	,993	,003	5	687
25	1,000	0	.	.	5	686
26	1,000	0	.	.	5	685
27	1,000	0	.	.	5	684
28	1,000	0	.	.	5	683
29	2,000	1	,991	,003	6	682
30	2,000	0	.	.	6	681
31	2,000	0	.	.	6	680
32	2,000	0	.	.	6	679
33	2,000	0	.	.	6	678
34	2,000	0	.	.	6	677
35	2,000	0	.	.	6	676
36	2,000	0	.	.	6	675
37	3,000	0	.	.	6	674
38	3,000	0	.	.	6	673
39	3,000	0	.	.	6	672
40	3,000	0	.	.	6	671
41	3,000	0	.	.	6	670
42	3,000	0	.	.	6	669
43	3,000	0	.	.	6	668
44	3,000	0	.	.	6	667
45	3,000	0	.	.	6	666
46	3,000	0	.	.	6	665
47	4,000	1	.	.	7	664

Überlebenstabelle

			Kumulierter Anteil Überlebender zum Zeitpunkt			
	Zeit	Status	Schätzer	Standardfehler	Anzahl der kumulativen Ereignisse	Anzahl der verbliebenen Fälle
48	4,000	1	.	.	8	663
49	4,000	1	.	.	9	662
50	4,000	1	,985	,005	10	661
51	4,000	0	.	.	10	660
52	4,000	0	.	.	10	659
53	4,000	0	.	.	10	658
54	4,000	0	.	.	10	657
55	4,000	0	.	.	10	656
56	4,000	0	.	.	10	655
57	4,000	0	.	.	10	654
58	4,000	0	.	.	10	653
59	4,000	0	.	.	10	652
60	4,000	0	.	.	10	651
61	4,000	0	.	.	10	650
62	5,000	0	.	.	10	649
63	5,000	0	.	.	10	648
64	5,000	0	.	.	10	647
65	5,000	0	.	.	10	646
66	5,000	0	.	.	10	645
67	5,000	0	.	.	10	644
68	5,000	0	.	.	10	643
69	5,000	0	.	.	10	642
70	5,000	0	.	.	10	641
71	5,000	0	.	.	10	640
72	5,000	0	.	.	10	639
73	5,000	0	.	.	10	638
74	5,000	0	.	.	10	637
75	5,000	0	.	.	10	636
76	5,000	0	.	.	10	635
77	5,000	0	.	.	10	634
78	6,000	1	.	.	11	633
79	6,000	1	.	.	12	632
80	6,000	1	.	.	13	631
81	6,000	1	,979	,005	14	630
82	6,000	0	.	.	14	629
83	6,000	0	.	.	14	628
84	6,000	0	.	.	14	627
85	6,000	0	.	.	14	626
86	6,000	0	.	.	14	625
87	6,000	0	.	.	14	624
88	6,000	0	.	.	14	623
89	6,000	0	.	.	14	622
90	6,000	0	.	.	14	621
91	6,000	0	.	.	14	620
92	6,000	0	.	.	14	619
93	6,000	0	.	.	14	618
94	6,000	0	.	.	14	617
95	6,000	0	.	.	14	616

Überlebenstabelle

	Zeit	Status	Kumulierter Anteil Überlebender zum Zeitpunkt		Anzahl der kumulativen Ereignisse	Anzahl der verbliebenen Fälle
			Schätzer	Standardfehler		
96	6,000	0	.	.	14	615
97	6,000	0	.	.	14	614
98	7,000	1	,978	,006	15	613
99	7,000	0	.	.	15	612
100	7,000	0	.	.	15	611
101	7,000	0	.	.	15	610
102	7,000	0	.	.	15	609
103	7,000	0	.	.	15	608
104	7,000	0	.	.	15	607
105	7,000	0	.	.	15	606
106	7,000	0	.	.	15	605
107	7,000	0	.	.	15	604
108	7,000	0	.	.	15	603
109	7,000	0	.	.	15	602
110	7,000	0	.	.	15	601
111	7,000	0	.	.	15	600
112	7,000	0	.	.	15	599
113	7,000	0	.	.	15	598
114	7,000	0	.	.	15	597
115	8,000	1	,976	,006	16	596
116	8,000	0	.	.	16	595
117	8,000	0	.	.	16	594
118	8,000	0	.	.	16	593
119	8,000	0	.	.	16	592
120	8,000	0	.	.	16	591
121	8,000	0	.	.	16	590
122	8,000	0	.	.	16	589
123	8,000	0	.	.	16	588
124	8,000	0	.	.	16	587
125	8,000	0	.	.	16	586
126	8,000	0	.	.	16	585
127	8,000	0	.	.	16	584
128	8,000	0	.	.	16	583
129	8,000	0	.	.	16	582
130	8,000	0	.	.	16	581
131	8,000	0	.	.	16	580
132	9,000	1	.	.	17	579
133	9,000	1	,973	,006	18	578
134	10,000	1	.	.	19	577
135	10,000	1	,969	,007	20	576
136	10,000	0	.	.	20	575
137	10,000	0	.	.	20	574
138	10,000	0	.	.	20	573
139	10,000	0	.	.	20	572
140	10,000	0	.	.	20	571
141	10,000	0	.	.	20	570
142	10,000	0	.	.	20	569
143	10,000	0	.	.	20	568

Überlebenstabelle

	Zeit	Status	Kumulierter Anteil Überlebender zum Zeitpunkt		Anzahl der kumulativen Ereignisse	Anzahl der verbliebenen Fälle
			Schätzer	Standardfehler		
144	10,000	0	.	.	20	567
145	10,000	0	.	.	20	566
146	10,000	0	.	.	20	565
147	10,000	0	.	.	20	564
148	10,000	0	.	.	20	563
149	10,000	0	.	.	20	562
150	10,000	0	.	.	20	561
151	11,000	1	.	.	21	560
152	11,000	1	.	.	22	559
153	11,000	1	.	.	23	558
154	11,000	1	,962	,008	24	557
155	11,000	0	.	.	24	556
156	11,000	0	.	.	24	555
157	11,000	0	.	.	24	554
158	11,000	0	.	.	24	553
159	11,000	0	.	.	24	552
160	11,000	0	.	.	24	551
161	11,000	0	.	.	24	550
162	11,000	0	.	.	24	549
163	11,000	0	.	.	24	548
164	11,000	0	.	.	24	547
165	11,000	0	.	.	24	546
166	11,000	0	.	.	24	545
167	11,000	0	.	.	24	544
168	11,000	0	.	.	24	543
169	11,000	0	.	.	24	542
170	11,000	0	.	.	24	541
171	12,000	0	.	.	24	540
172	12,000	0	.	.	24	539
173	12,000	0	.	.	24	538
174	12,000	0	.	.	24	537
175	12,000	0	.	.	24	536
176	12,000	0	.	.	24	535
177	12,000	0	.	.	24	534
178	12,000	0	.	.	24	533
179	12,000	0	.	.	24	532
180	12,000	0	.	.	24	531
181	12,000	0	.	.	24	530
182	12,000	0	.	.	24	529
183	12,000	0	.	.	24	528
184	12,000	0	.	.	24	527
185	12,000	0	.	.	24	526
186	12,000	0	.	.	24	525
187	12,000	0	.	.	24	524
188	12,000	0	.	.	24	523
189	14,000	1	.	.	25	522
190	14,000	1	.	.	26	521
191	14,000	1	.	.	27	520

Überlebenstabelle

	Zeit	Status	Kumulierter Anteil Überlebender zum Zeitpunkt		Anzahl der kumulativen Ereignisse	Anzahl der verbliebenen Fälle
			Schätzer	Standardfehler		
192	14,000	1	.	.	28	519
193	14,000	1	,953	,009	29	518
194	15,000	1	,951	,009	30	517
195	15,000	0	.	.	30	516
196	15,000	0	.	.	30	515
197	15,000	0	.	.	30	514
198	15,000	0	.	.	30	513
199	15,000	0	.	.	30	512
200	15,000	0	.	.	30	511
201	15,000	0	.	.	30	510
202	15,000	0	.	.	30	509
203	15,000	0	.	.	30	508
204	17,000	1	.	.	31	507
205	17,000	1	,948	,009	32	506
206	17,000	0	.	.	32	505
207	17,000	0	.	.	32	504
208	17,000	0	.	.	32	503
209	17,000	0	.	.	32	502
210	17,000	0	.	.	32	501
211	17,000	0	.	.	32	500
212	17,000	0	.	.	32	499
213	17,000	0	.	.	32	498
214	17,000	0	.	.	32	497
215	19,000	0	.	.	32	496
216	19,000	0	.	.	32	495
217	19,000	0	.	.	32	494
218	19,000	0	.	.	32	493
219	20,000	0	.	.	32	492
220	20,000	0	.	.	32	491
221	20,000	0	.	.	32	490
222	20,000	0	.	.	32	489
223	20,000	0	.	.	32	488
224	20,000	0	.	.	32	487
225	20,000	0	.	.	32	486
226	20,000	0	.	.	32	485
227	20,000	0	.	.	32	484
228	20,000	0	.	.	32	483
229	21,000	0	.	.	32	482
230	21,000	0	.	.	32	481
231	21,000	0	.	.	32	480
232	21,000	0	.	.	32	479
233	21,000	0	.	.	32	478
234	21,000	0	.	.	32	477
235	21,000	0	.	.	32	476
236	21,000	0	.	.	32	475
237	22,000	1	,946	,009	33	474
238	22,000	0	.	.	33	473
239	22,000	0	.	.	33	472

Überlebenstabelle

	Zeit	Status	Kumulierter Anteil Überlebender zum Zeitpunkt		Anzahl der kumulativen Ereignisse	Anzahl der verbliebenen Fälle
			Schätzer	Standardfehler		
240	22,000	0	.	.	33	471
241	22,000	0	.	.	33	470
242	22,000	0	.	.	33	469
243	22,000	0	.	.	33	468
244	22,000	0	.	.	33	467
245	22,000	0	.	.	33	466
246	22,000	0	.	.	33	465
247	22,000	0	.	.	33	464
248	23,000	1	.	.	34	463
249	23,000	1	.	.	35	462
250	23,000	1	,939	,010	36	461
251	23,000	0	.	.	36	460
252	23,000	0	.	.	36	459
253	23,000	0	.	.	36	458
254	24,000	1	,937	,010	37	457
255	24,000	0	.	.	37	456
256	24,000	0	.	.	37	455
257	24,000	0	.	.	37	454
258	24,000	0	.	.	37	453
259	24,000	0	.	.	37	452
260	24,000	0	.	.	37	451
261	24,000	0	.	.	37	450
262	24,000	0	.	.	37	449
263	24,000	0	.	.	37	448
264	24,000	0	.	.	37	447
265	24,000	0	.	.	37	446
266	24,000	0	.	.	37	445
267	24,000	0	.	.	37	444
268	25,000	1	,935	,010	38	443
269	25,000	0	.	.	38	442
270	25,000	0	.	.	38	441
271	25,000	0	.	.	38	440
272	26,000	1	.	.	39	439
273	26,000	1	.	.	40	438
274	26,000	1	,929	,011	41	437
275	26,000	0	.	.	41	436
276	26,000	0	.	.	41	435
277	26,000	0	.	.	41	434
278	26,000	0	.	.	41	433
279	26,000	0	.	.	41	432
280	26,000	0	.	.	41	431
281	26,000	0	.	.	41	430
282	26,000	0	.	.	41	429
283	26,000	0	.	.	41	428
284	26,000	0	.	.	41	427
285	26,000	0	.	.	41	426
286	26,000	0	.	.	41	425
287	26,000	0	.	.	41	424

Überlebenstabelle

			Kumulierter Anteil Überlebender zum Zeitpunkt		Anzahl der kumulativen Ereignisse	Anzahl der verbliebenen Fälle
	Zeit	Status	Schätzer	Standardfehler		
288	26,000	0	.	.	41	423
289	26,000	0	.	.	41	422
290	26,000	0	.	.	41	421
291	26,000	0	.	.	41	420
292	27,000	0	.	.	41	419
293	27,000	0	.	.	41	418
294	27,000	0	.	.	41	417
295	27,000	0	.	.	41	416
296	27,000	0	.	.	41	415
297	27,000	0	.	.	41	414
298	27,000	0	.	.	41	413
299	27,000	0	.	.	41	412
300	27,000	0	.	.	41	411
301	27,000	0	.	.	41	410
302	28,000	0	.	.	41	409
303	28,000	0	.	.	41	408
304	28,000	0	.	.	41	407
305	28,000	0	.	.	41	406
306	28,000	0	.	.	41	405
307	28,000	0	.	.	41	404
308	28,000	0	.	.	41	403
309	28,000	0	.	.	41	402
310	28,000	0	.	.	41	401
311	28,000	0	.	.	41	400
312	28,000	0	.	.	41	399
313	28,000	0	.	.	41	398
314	29,000	1	.	.	42	397
315	29,000	1	.	.	43	396
316	29,000	1	.	.	44	395
317	29,000	1	,920	,012	45	394
318	29,000	0	.	.	45	393
319	29,000	0	.	.	45	392
320	29,000	0	.	.	45	391
321	29,000	0	.	.	45	390
322	29,000	0	.	.	45	389
323	30,000	1	.	.	46	388
324	30,000	1	.	.	47	387
325	30,000	1	.	.	48	386
326	30,000	1	,910	,012	49	385
327	31,000	0	.	.	49	384
328	31,000	0	.	.	49	383
329	31,000	0	.	.	49	382
330	31,000	0	.	.	49	381
331	31,000	0	.	.	49	380
332	31,000	0	.	.	49	379
333	31,000	0	.	.	49	378
334	31,000	0	.	.	49	377
335	31,000	0	.	.	49	376

Überlebenstabelle

	Zeit	Status	Kumulierter Anteil Überlebender zum Zeitpunkt		Anzahl der kumulativen Ereignisse	Anzahl der verbliebenen Fälle
			Schätzer	Standardfehler		
336	31,000	0	.	.	49	375
337	31,000	0	.	.	49	374
338	31,000	0	.	.	49	373
339	31,000	0	.	.	49	372
340	31,000	0	.	.	49	371
341	31,000	0	.	.	49	370
342	31,000	0	.	.	49	369
343	32,000	0	.	.	49	368
344	32,000	0	.	.	49	367
345	32,000	0	.	.	49	366
346	32,000	0	.	.	49	365
347	32,000	0	.	.	49	364
348	32,000	0	.	.	49	363
349	32,000	0	.	.	49	362
350	33,000	1	.	.	50	361
351	33,000	1	.	.	51	360
352	33,000	1	,903	,013	52	359
353	33,000	0	.	.	52	358
354	33,000	0	.	.	52	357
355	33,000	0	.	.	52	356
356	33,000	0	.	.	52	355
357	34,000	0	.	.	52	354
358	34,000	0	.	.	52	353
359	34,000	0	.	.	52	352
360	34,000	0	.	.	52	351
361	34,000	0	.	.	52	350
362	34,000	0	.	.	52	349
363	35,000	0	.	.	52	348
364	36,000	1	,900	,013	53	347
365	36,000	0	.	.	53	346
366	36,000	0	.	.	53	345
367	36,000	0	.	.	53	344
368	36,000	0	.	.	53	343
369	36,000	0	.	.	53	342
370	36,000	0	.	.	53	341
371	36,000	0	.	.	53	340
372	36,000	0	.	.	53	339
373	36,000	0	.	.	53	338
374	36,000	0	.	.	53	337
375	37,000	0	.	.	53	336
376	37,000	0	.	.	53	335
377	37,000	0	.	.	53	334
378	37,000	0	.	.	53	333
379	37,000	0	.	.	53	332
380	38,000	1	.	.	54	331
381	38,000	1	,895	,014	55	330
382	38,000	0	.	.	55	329
383	38,000	0	.	.	55	328

Überlebenstabelle

	Zeit	Status	Kumulierter Anteil Überlebender zum Zeitpunkt		Anzahl der kumulativen Ereignisse	Anzahl der verbliebenen Fälle
			Schätzer	Standardfehler		
384	38,000	0	.	.	55	327
385	38,000	0	.	.	55	326
386	38,000	0	.	.	55	325
387	38,000	0	.	.	55	324
388	39,000	0	.	.	55	323
389	40,000	0	.	.	55	322
390	40,000	0	.	.	55	321
391	40,000	0	.	.	55	320
392	40,000	0	.	.	55	319
393	40,000	0	.	.	55	318
394	40,000	0	.	.	55	317
395	40,000	0	.	.	55	316
396	41,000	1	.	.	56	315
397	41,000	1	,889	,014	57	314
398	41,000	0	.	.	57	313
399	41,000	0	.	.	57	312
400	41,000	0	.	.	57	311
401	41,000	0	.	.	57	310
402	41,000	0	.	.	57	309
403	41,000	0	.	.	57	308
404	41,000	0	.	.	57	307
405	41,000	0	.	.	57	306
406	42,000	0	.	.	57	305
407	42,000	0	.	.	57	304
408	42,000	0	.	.	57	303
409	42,000	0	.	.	57	302
410	42,000	0	.	.	57	301
411	42,000	0	.	.	57	300
412	42,000	0	.	.	57	299
413	42,000	0	.	.	57	298
414	42,000	0	.	.	57	297
415	44,000	0	.	.	57	296
416	44,000	0	.	.	57	295
417	44,000	0	.	.	57	294
418	44,000	0	.	.	57	293
419	44,000	0	.	.	57	292
420	44,000	0	.	.	57	291
421	45,000	0	.	.	57	290
422	45,000	0	.	.	57	289
423	46,000	0	.	.	57	288
424	47,000	0	.	.	57	287
425	47,000	0	.	.	57	286
426	47,000	0	.	.	57	285
427	47,000	0	.	.	57	284
428	47,000	0	.	.	57	283
429	48,000	1	,886	,015	58	282
430	49,000	0	.	.	58	281
431	50,000	0	.	.	58	280

Überlebenstabelle

			Kumulierter Anteil Überlebender zum Zeitpunkt			
	**Zeit**	**Status**	**Schätzer**	Standardfehler	Anzahl der kumulativen Ereignisse	Anzahl der verbliebenen Fälle
432	50,000	0	.	.	58	279
433	50,000	0	.	.	58	278
434	51,000	0	.	.	58	277
435	51,000	0	.	.	58	276
436	51,000	0	.	.	58	275
437	51,000	0	.	.	58	274
438	51,000	0	.	.	58	273
439	51,000	0	.	.	58	272
440	51,000	0	.	.	58	271
441	51,000	0	.	.	58	270
442	51,000	0	.	.	58	269
443	51,000	0	.	.	58	268
444	51,000	0	.	.	58	267
445	51,000	0	.	.	58	266
446	51,000	0	.	.	58	265
447	51,000	0	.	.	58	264
448	51,000	0	.	.	58	263
449	51,000	0	.	.	58	262
450	51,000	0	.	.	58	261
451	51,000	0	.	.	58	260
452	51,000	0	.	.	58	259
453	51,000	0	.	.	58	258
454	51,000	0	.	.	58	257
455	52,000	0	.	.	58	256
456	52,000	0	.	.	58	255
457	52,000	0	.	.	58	254
458	52,000	0	.	.	58	253
459	53,000	0	.	.	58	252
460	53,000	0	.	.	58	251
461	53,000	0	.	.	58	250
462	53,000	0	.	.	58	249
463	53,000	0	.	.	58	248
464	53,000	0	.	.	58	247
465	53,000	0	.	.	58	246
466	53,000	0	.	.	58	245
467	53,000	0	.	.	58	244
468	53,000	0	.	.	58	243
469	53,000	0	.	.	58	242
470	53,000	0	.	.	58	241
471	53,000	0	.	.	58	240
472	53,000	0	.	.	58	239
473	53,000	0	.	.	58	238
474	53,000	0	.	.	58	237
475	54,000	0	.	.	58	236
476	54,000	0	.	.	58	235
477	54,000	0	.	.	58	234
478	54,000	0	.	.	58	233
479	55,000	0	.	.	58	232

Überlebenstabelle

	Zeit	Status	Kumulierter Anteil Überlebender zum Zeitpunkt		Anzahl der kumulativen Ereignisse	Anzahl der verbliebenen Fälle
			Schätzer	Standardfehler		
480	55,000	0	.	.	58	231
481	55,000	0	.	.	58	230
482	55,000	0	.	.	58	229
483	55,000	0	.	.	58	228
484	55,000	0	.	.	58	227
485	55,000	0	.	.	58	226
486	56,000	0	.	.	58	225
487	56,000	0	.	.	58	224
488	56,000	0	.	.	58	223
489	56,000	0	.	.	58	222
490	56,000	0	.	.	58	221
491	56,000	0	.	.	58	220
492	56,000	0	.	.	58	219
493	56,000	0	.	.	58	218
494	56,000	0	.	.	58	217
495	56,000	0	.	.	58	216
496	57,000	0	.	.	58	215
497	57,000	0	.	.	58	214
498	58,000	0	.	.	58	213
499	58,000	0	.	.	58	212
500	58,000	0	.	.	58	211
501	58,000	0	.	.	58	210
502	58,000	0	.	.	58	209
503	58,000	0	.	.	58	208
504	58,000	0	.	.	58	207
505	59,000	0	.	.	58	206
506	59,000	0	.	.	58	205
507	59,000	0	.	.	58	204
508	59,000	0	.	.	58	203
509	59,000	0	.	.	58	202
510	59,000	0	.	.	58	201
511	59,000	0	.	.	58	200
512	60,000	1	.	.	59	199
513	60,000	1	.	.	60	198
514	60,000	1	,873	,016	61	197
515	61,000	0	.	.	61	196
516	61,000	0	.	.	61	195
517	61,000	0	.	.	61	194
518	61,000	0	.	.	61	193
519	61,000	0	.	.	61	192
520	61,000	0	.	.	61	191
521	62,000	0	.	.	61	190
522	62,000	0	.	.	61	189
523	62,000	0	.	.	61	188
524	62,000	0	.	.	61	187
525	63,000	0	.	.	61	186
526	63,000	0	.	.	61	185
527	64,000	0	.	.	61	184

Überlebenstabelle

			Kumulierter Anteil Überlebender zum Zeitpunkt			
	Zeit	Status	Schätzer	Standardfehler	Anzahl der kumulativen Ereignisse	Anzahl der verbliebenen Fälle
528	64,000	0	.	.	61	183
529	65,000	0	.	.	61	182
530	65,000	0	.	.	61	181
531	65,000	0	.	.	61	180
532	65,000	0	.	.	61	179
533	66,000	1	,868	,017	62	178
534	66,000	0	.	.	62	177
535	66,000	0	.	.	62	176
536	66,000	0	.	.	62	175
537	66,000	0	.	.	62	174
538	67,000	0	.	.	62	173
539	67,000	0	.	.	62	172
540	67,000	0	.	.	62	171
541	67,000	0	.	.	62	170
542	67,000	0	.	.	62	169
543	67,000	0	.	.	62	168
544	67,000	0	.	.	62	167
545	67,000	0	.	.	62	166
546	68,000	0	.	.	62	165
547	68,000	0	.	.	62	164
548	68,000	0	.	.	62	163
549	68,000	0	.	.	62	162
550	68,000	0	.	.	62	161
551	70,000	1	.	.	63	160
552	70,000	1	,857	,018	64	159
553	70,000	0	.	.	64	158
554	71,000	1	,851	,019	65	157
555	72,000	0	.	.	65	156
556	72,000	0	.	.	65	155
557	72,000	0	.	.	65	154
558	72,000	0	.	.	65	153
559	73,000	1	,846	,020	66	152
560	73,000	0	.	.	66	151
561	73,000	0	.	.	66	150
562	74,000	0	.	.	66	149
563	74,000	0	.	.	66	148
564	75,000	0	.	.	66	147
565	76,000	0	.	.	66	146
566	76,000	0	.	.	66	145
567	78,000	0	.	.	66	144
568	83,000	0	.	.	66	143
569	83,000	0	.	.	66	142
570	83,000	0	.	.	66	141
571	83,000	0	.	.	66	140
572	83,000	0	.	.	66	139
573	83,000	0	.	.	66	138
574	83,000	0	.	.	66	137
575	83,000	0	.	.	66	136

Überlebenstabelle

	Zeit	Status	Kumulierter Anteil Überlebender zum Zeitpunkt		Anzahl der kumulativen Ereignisse	Anzahl der verbliebenen Fälle
			Schätzer	Standardfehler		
576	84,000	0	.	.	66	135
577	84,000	0	.	.	66	134
578	85,000	0	.	.	66	133
579	85,000	0	.	.	66	132
580	85,000	0	.	.	66	131
581	86,000	1	,839	,021	67	130
582	86,000	0	.	.	67	129
583	87,000	0	.	.	67	128
584	87,000	0	.	.	67	127
585	88,000	0	.	.	67	126
586	88,000	0	.	.	67	125
587	88,000	0	.	.	67	124
588	88,000	0	.	.	67	123
589	89,000	0	.	.	67	122
590	89,000	0	.	.	67	121
591	90,000	0	.	.	67	120
592	90,000	0	.	.	67	119
593	90,000	0	.	.	67	118
594	90,000	0	.	.	67	117
595	93,000	0	.	.	67	116
596	93,000	0	.	.	67	115
597	93,000	0	.	.	67	114
598	93,000	0	.	.	67	113
599	93,000	0	.	.	67	112
600	93,000	0	.	.	67	111
601	93,000	0	.	.	67	110
602	93,000	0	.	.	67	109
603	94,000	0	.	.	67	108
604	94,000	0	.	.	67	107
605	95,000	0	.	.	67	106
606	95,000	0	.	.	67	105
607	95,000	0	.	.	67	104
608	95,000	0	.	.	67	103
609	95,000	0	.	.	67	102
610	95,000	0	.	.	67	101
611	95,000	0	.	.	67	100
612	95,000	0	.	.	67	99
613	96,000	0	.	.	67	98
614	96,000	0	.	.	67	97
615	96,000	0	.	.	67	96
616	96,000	0	.	.	67	95
617	96,000	0	.	.	67	94
618	96,000	0	.	.	67	93
619	96,000	0	.	.	67	92
620	96,000	0	.	.	67	91
621	96,000	0	.	.	67	90
622	97,000	0	.	.	67	89
623	97,000	0	.	.	67	88

Überlebenstabelle

	Zeit	Status	Kumulierter Anteil Überlebender zum Zeitpunkt		Anzahl der kumulativen Ereignisse	Anzahl der verbliebenen Fälle
			Schätzer	Standardfehler		
624	99,000	0	.	.	67	87
625	99,000	0	.	.	67	86
626	99,000	0	.	.	67	85
627	99,000	0	.	.	67	84
628	99,000	0	.	.	67	83
629	99,000	0	.	.	67	82
630	99,000	0	.	.	67	81
631	101,000	0	.	.	67	80
632	101,000	0	.	.	67	79
633	101,000	0	.	.	67	78
634	101,000	0	.	.	67	77
635	101,000	0	.	.	67	76
636	101,000	0	.	.	67	75
637	101,000	0	.	.	67	74
638	103,000	0	.	.	67	73
639	103,000	0	.	.	67	72
640	103,000	0	.	.	67	71
641	103,000	0	.	.	67	70
642	103,000	0	.	.	67	69
643	103,000	0	.	.	67	68
644	103,000	0	.	.	67	67
645	103,000	0	.	.	67	66
646	105,000	0	.	.	67	65
647	110,000	1	.	.	68	64
648	110,000	1	,814	,027	69	63
649	110,000	0	.	.	69	62
650	110,000	0	.	.	69	61
651	110,000	0	.	.	69	60
652	110,000	0	.	.	69	59
653	110,000	0	.	.	69	58
654	111,000	1	,800	,030	70	57
655	113,000	0	.	.	70	56
656	113,000	0	.	.	70	55
657	113,000	0	.	.	70	54
658	113,000	0	.	.	70	53
659	116,000	0	.	.	70	52
660	116,000	0	.	.	70	51
661	116,000	0	.	.	70	50
662	116,000	0	.	.	70	49
663	116,000	0	.	.	70	48
664	116,000	0	.	.	70	47
665	118,000	0	.	.	70	46
666	118,000	0	.	.	70	45
667	119,000	0	.	.	70	44
668	119,000	0	.	.	70	43
669	119,000	0	.	.	70	42
670	119,000	0	.	.	70	41
671	119,000	0	.	.	70	40

Überlebenstabelle

	Zeit	Status	Kumulierter Anteil Überlebender zum Zeitpunkt		Anzahl der kumulativen Ereignisse	Anzahl der verbliebenen Fälle
			Schätzer	Standardfehler		
672	119,000	0	.	.	70	39
673	119,000	0	.	.	70	38
674	119,000	0	.	.	70	37
675	119,000	0	.	.	70	36
676	119,000	0	.	.	70	35
677	119,000	0	.	.	70	34
678	120,000	0	.	.	70	33
679	120,000	0	.	.	70	32
680	120,000	0	.	.	70	31
681	120,000	0	.	.	70	30
682	120,000	0	.	.	70	29
683	120,000	0	.	.	70	28
684	120,000	0	.	.	70	27
685	120,000	0	.	.	70	26
686	124,000	0	.	.	70	25
687	124,000	0	.	.	70	24
688	132,000	0	.	.	70	23
689	132,000	0	.	.	70	22
690	132,000	0	.	.	70	21
691	132,000	0	.	.	70	20
692	132,000	0	.	.	70	19
693	132,000	0	.	.	70	18
694	139,000	0	.	.	70	17
695	139,000	0	.	.	70	16
696	139,000	0	.	.	70	15
697	147,000	0	.	.	70	14
698	147,000	0	.	.	70	13
699	147,000	0	.	.	70	12
700	147,000	0	.	.	70	11
701	157,000	0	.	.	70	10
702	157,000	0	.	.	70	9
703	157,000	0	.	.	70	8
704	158,000	0	.	.	70	7
705	158,000	0	.	.	70	6
706	158,000	0	.	.	70	5
707	158,000	0	.	.	70	4
708	227,000	0	.	.	70	3
709	227,000	0	.	.	70	2
710	227,000	0	.	.	70	1
711	227,000	0	.	.	70	0
